# General Surgery

**Published:** 1895-11-30

**Authors:** 


### PROGRESS IN SURGERY.

### GENERAL SURGERY.

**Fractures and Dislocations.**—In a recent lecture on fracture of the long bones from slight causes W. H. Bennett[1] gives the following as the principal conditions which tend to easy fracture—(1) General: locomotor ataxy, rickets, mollities ossium, fragilitas ossium, general fatty degeneration, chronic lead poisoning, malignant disease. (2) Local: bone atrophy from long disuse, from injury to the nutrient artery (?), in old age (intracapsular fracture of femur), weakness of the bone from necrosis or caries, malignant diseases. (3) Muscular action alone may cause fracture under certain circumstances. Bennett describes a phenomenal illustration of multiple fractures from slight causes, the brittleness of the bones depending upon chronic lead poisoning, which is hardly recognised as it should be as a cause of this condition, and mentions an extremely useful and practical point, viz., that when a patient, who is concerned, in his work or in other ways, with lead, suffers from fracture, a fortnight, three weeks, or perhaps even a month longer should be allowed to bring about a thoroughly sound union than should be given another man whose occupation does not bring him in contact with lead. He concludes with the following practical deductions: (1) Fractures from slight causes connected with (*a*) tabes and locomotor ataxy unite rapidly but not strongly; (*b*) rickets, fragilitas ossium, and lead poisoning unite well but sometimes slowly; (*c*) malignant disease very rarely unite at all, and the union if it occurs soon breaks down again; (*d*) mollities ossium and general fatty degeneration do not unite at all; (*e*) fractures of long bones caused by muscular action alone unite well and strongly. (2) Fractures from slight causes are for practical purposes always transverse; they are, therefore, very easily managed. (3) All cases of fracture in which the violence described appears inadequate for the production of the lesion should be regarded with suspicion, and a very careful examination of the patient made, in order to ascertain whether either of the conditions enumerated above is present. (4) Fractures from slight causes are rarely if ever seen in limbs affected by infantile paralysis or other forms of palsy connected with disease of the brain or spinal cord. From Jay Gould's[2] experience in injury to bone tissue by the Lee-Metford bullet, he thinks it causes but slight shock, and doubts its capacity of putting a man out of action. Instead of badly fracturing and comminuting the bone, it appears only to drill the bone and slightly splinter the edges. A hard bone like the shaft of the femur, it in all probability would fracture if it hit it fair, otherwise it would groove and glance off it. It apparently has little comminuting action even on the skull bones. H. Knaggs[3] records a case demonstrating gunshot injuries produced by the same weapon. As regards the injury to the third lumbar vertebra, though a considerable portion of its body was destroyed, the wound of exit was hardly larger than the wound of entrance, which is unusual in such cases, especially at short ranges, where the exit of the bullet is often marked by a wound of great extent. The Bier method of treating tuberculous joint affections by passive congestion has, according to F. M. Caird,[4] been formerly employed by Dumreicher for the cure of ununited fractures. This mode of treatment, although apparently well known on the Continent, is somewhat novel here, and the two cases of ununited fracture of the femur which Caird describes, serve to illustrate the advantage gained by the simple means recommended, namely, the use of a carefully-applied flannel roller and an Esmarch's tourniquet. C. J. Bond[5] is of opinion that deformed and partially useless limbs, the result of previous injury, should be treated by more decided and more scientific action by the operating surgeon (the other side of bone setting). He gives cases illustrating some of the results of old injury on different joints, and segments of the two extremities, which show the value of operative treatment. Douglas Graham[6] says that in a recent acute sprain the skilled masseur does not begin at once to masser an injured joint, but at a distance above it on the healthy tissues, by gently stroking, effleurage, towards the heart, gradually proceeding nearer and nearer to the painful place. This has a soothing effect, and pushes the flow along in the veins and lymphatics, making more space in them for the returning currents coming from beyond the region worked upon, and carrying away the fluids that have leaked out of the vessels. The same should be done on the part of the limb beyond the joint, for the circulation is hindered both in going out and coming in by reason of the swelling. The masseur begins again at a safe distance above the injured joint, and uses deep rubbing, kneading, or massage properly so-called, one hand contracting as the other relaxes, alternately making circular grasps, and this should be done on the parts above and below the seat of the sprain. By this procedure the effects of the previous effleurage are much enhanced, an analgesic or agreeably benumbing effect is produced upon the nerves, which extends to the painful place, and the retarded circulation is pushed along more vigorously, making room in the vessels for the swelling, the effusion, of blood and lymph further up the stream to float off. At the end of fifteen or twenty minutes of this manner of working, gentle, firm pressure can be made immediately over the swollen and but recently very tender parts, which, in a few seconds, can have circular motion, with the greatest push upwards added to it; and this, if sufficient tact be used, will in all probability not hurt, but be positively agreeable. A well-fitting bandage should then be applied. Under this plan of treatment, used twice a day, the comfort produced and the speed of recovery would scarcely be believed unless experienced by one who had a similar injury treated in the regular orthodox way, with absolute rest and immobility by means of fixed dressings.

[1] Clinical Journal, Feb. 27, 1895, p. 277. [2] Brit. Med. J., July 20, 1895, p. 129. [3] Ibid, p. 131. [4] Edin. Med. Journal, June, 1895, p. 1,078. [5] Provincial Med. Journal, March 1, 1895, p. 131. [6] Edin. Med. Journal, Aug., 1895, p. 122.

